# Surveillance of Respiratory Pathogens Among Rapid Diagnostic Test-Negative Acute Respiratory Infection Patients in Myanmar in 2023, with a Focus on Rhinovirus and Enterovirus Genotyping

**DOI:** 10.3390/v17060860

**Published:** 2025-06-17

**Authors:** Yuyang Sun, Tsutomu Tamura, Yadanar Kyaw, Swe Setk, Moe Myat Aye, Htay Htay Tin, Su Mon Kyaw Win, Jiaming Li, Tri Bayu Purnama, Irina Chon, Keita Wagatsuma, Hisami Watanabe, Reiko Saito

**Affiliations:** 1 Division of International Health (Public Health), Graduate School of Medical and Dental Sciences, Niigata University, Niigata 951-8510, Japanjasmine@med.niigata-u.ac.jp (R.S.); 2 Infectious Diseases Research Center of Niigata University in Myanmar, Niigata University, Niigata 951-8510, Japan; 3 Respiratory Medicine Department, Thingangyun Sanpya General Hospital, Yangon 110-71, Myanmar; 4 National Health Laboratory, Department of Medical Services, Dagon Township, Yangon 111-91, Myanmar; 5 University of Medical Technology, Yangon 110-12, Myanmar

**Keywords:** rhinovirus, enterovirus, molecular epidemiology, acute respiratory infection, COVID-19 pandemic

## Abstract

This study explored the distribution and genetic characteristics of respiratory pathogens in outpatients with acute respiratory infections (ARIs) in Yangon, Myanmar, during the 2023 rainy season. Among 267 patients who tested negative for influenza, RSV, and SARS-CoV-2 using rapid diagnostic tests, 84.6% were positive for at least one pathogen according to a multiplex polymerase chain reaction (PCR) assay, the BioFire^®^ FilmArray^®^ Respiratory Panel 2.1. The most common viruses detected were rhinovirus/enterovirus (RV/EV) at 37.8%, respiratory syncytial virus (RSV) at 22.4%, and human metapneumovirus (hMPV) at 10.0%. These pathogens co-circulated mainly from July to September, with RV/EV consistently predominant. Symptom comparison among RV/EV-, RSV-, and hMPV-infected patients showed similar clinical features, though fever was more common in hMPV cases. Among RV/EV-positive patients, 59.3% had single infections, while 40.7% experienced co-infections, especially with RSV and adenovirus. Genotyping identified 28 types from five species, primarily RV-A and RV-C, which were genetically diverse. One EV-D68 case was also found, emphasizing its potential risk. This study underscores the genetic diversity and clinical impact of RV/EV and stresses the importance of ongoing molecular surveillance in Myanmar’s post-COVID-19 context to inform effective public health responses.

## 1. Introduction

Acute respiratory tract infection (ARI) is a major cause of acute illness worldwide, characterized by high morbidity and high infectiousness, and is the most prominent cause of childhood mortality [1,2,3,4]. Approximately 15% of global under-5 mortality is attributed to ARI, with the majority of cases occurring in low- and middle-income countries [5]. A recent global study showed that economic disparities and inequality are attributed to the increased ARI risk among these countries [6].

In contrast to the well-defined winter peaks observed in temperate regions, influenza, respiratory syncytial virus (RSV), and other respiratory viruses in tropical countries exhibit diverse seasonal patterns—ranging from annual or semiannual peaks to year-round low-level transmission—often influenced by local climatic factors such as humidity and rainfall, particularly during the rainy season [7,8,9]. The COVID-19 pandemic, which began in 2020, profoundly altered the epidemiology of respiratory viruses due to the widespread implementation of non-pharmaceutical interventions (NPIs), including lockdowns, social distancing, and mask-wearing. These measures effectively reduced the transmission of SARS-CoV-2 and also suppressed the circulation of many other respiratory viruses [10,11,12,13]. However, studies showed that rhinoviruses (RVs) were relatively unaffected by NPIs and continued to circulate throughout the pandemic. This observation might reflect the inherent environmental stability and resistance to standard control measures of these viruses, potentially compounded by complex virus–virus interactions influencing their transmission dynamics [14,15]. As NPIs were relaxed in 2022 and 2023, suppressed viruses such as influenza and respiratory syncytial virus re-emerged in many countries [11,13,16].

Over the past two decades, we have collaborated with researchers in Myanmar to conduct comprehensive surveillance and molecular epidemiological studies of respiratory viruses. Our outpatient surveillance conducted before the COVID-19 pandemic clarified the unique seasonal patterns of influenza in tropical Myanmar, where virus activity typically peaks during the rainy season from June to August, contrasting with the winter epidemics seen in temperate regions [17,18]. In addition, our study showed that RSV circulation in Myanmar also aligns with the rainy season [19]. In parallel, other respiratory viruses—including human metapneumovirus, enterovirus/rhinovirus, parainfluenza viruses (types 1–3), seasonal coronaviruses, adenovirus, bocavirus, parechovirus, and enterovirus D68—have also been investigated in Myanmar, primarily in hospitalized pediatric patients with acute lower respiratory infections [20,21,22]. However, the epidemiology of these respiratory viruses in outpatient settings remains poorly understood in Myanmar, highlighting the need for extend surveillance in mild ARI outpatients.

Identifying respiratory pathogens presents unique challenges, particularly in resource-limited contexts. The BioFire^®^ FilmArray^®^ Respiratory Panel 2.1 (herein BioFire RP; BioFire Diagnostics, Salt Lake City, UT, USA) offers a comprehensive and efficient solution. This fully automated multiplex PCR assay detects nucleic acids from 22 respiratory pathogens within one hour using a single nasopharyngeal swab. Target pathogens include adenovirus, SARS-CoV-2, coronaviruses-229E, coronaviruses-HKU1, coronaviruses-NL63, coronaviruses-OC43, human metapneumovirus (hMPV), RV/EV, influenza virus A, influenza virus A(H1N1), influenza virus A(H1N1)pdm09, influenza virus A(H3N2), influenza virus B, parainfluenza virus 1, parainfluenza virus 2, parainfluenza virus 3, parainfluenza virus 4, RSV, *Bordetella pertussis*, *Chlamydia pneumoniae*, and *Mycoplasma pneumoniae* [23,24]. 

Enteroviruses and rhinoviruses (EVs/RVs) are both members of the *Picornaviridae* family and are now classified within the same genus, Enterovirus, based on genomic and phylogenetic analyses. This genus includes multiple species, but only seven species (EV A–D and RV A–C) can cause human infection [25]. Although RVs were once considered to cause only mild upper respiratory infections, recent studies have underscored their substantial involvement in more severe clinical outcomes, including pneumonia, acute exacerbations of chronic obstructive pulmonary disease (COPD), and asthma exacerbations in pediatric populations [25,26,27,28]. Enteroviruses have a wider spectrum of pathogenicity and may cause serious diseases, although most infections are asymptomatic, such as poliomyelitis, encephalitis, aseptic meningitis, myocarditis, pericarditis, Reye’s syndrome, hand, foot and mouth disease (HFMD), conjunctivitis, uveitis, gastroenteritis, hepatitis, arthritis, and pancreatitis [25,28,29]. While RVs and EVs share a common genomic structure, the analysis of key gene regions—particularly VP4/VP2 and VP1—is essential for molecular typing and epidemiological studies. These regions provide insight into the viruses’ genetic diversity, evolutionary dynamics, and associated disease burden [30,31].

This study aimed to characterize the epidemiology of respiratory pathogens among outpatients with acute respiratory infections in Myanmar between June and December 2023. We focused on individuals who tested negative for influenza, RSV, and SARS-CoV-2 using rapid diagnostic tests, and analyzed their respiratory samples with the BioFire RP. As RVs/EVs emerged as the most frequently detected pathogens, we further conducted a comparative analysis of clinical manifestations, along with genotyping and phylogenetic characterization of RV/EV-positive samples to investigate their genetic diversity, temporal distribution, and co-infection patterns. These findings enhance our understanding of respiratory virus circulation in the post-COVID-19 era and underscore the need for ongoing molecular surveillance in outpatient settings, particularly in resource-limited areas. 

## 2. Materials and Methods

### 2.1. Study Location and Sample Collection from Participants

This observational study was conducted at the outpatient clinics of Thinganguyn General Hospital and Yankin Children’s Hospital in Yangon, Myanmar, between June and December 2023, and involved patients who presented with symptoms and signs of acute respiratory infection such as fever (≥37.5 °C), cough, wheezing, rhinorrhea, headache, dyspnea, myalgia, arthralgia, diarrhea, sore throat, sneezing, fatigue, nausea and vomiting, loss of smell, and loss of taste. Due to the outpatient nature of the study and lack of hospitalization data, we were unable to evaluate clinical severity or outcomes. The clinicians obtained informed consent from all study participants, including their parents and guardians. Patient information, including sex, age, and clinical symptoms, was recorded by the clinician. This study was approved by the Niigata University Ethical Committee (No. 2015-2533 and 2020-0155) and the Review Committee of the Department of Medical Research, Ministry of Health, Myanmar (No. 016516 and 2023-16). In this study, two nasal swabs were collected from each patient. One swab was used on site to screen for influenza A, influenza B, SARS-CoV-2, and RSV using RDTs (Quick-Navi™ Flu+COVID19Ag and Quick-Navi™ RSV2; Denka, Tokyo, Japan). The second swab was preserved in a viral transport medium and stored at −80 °C at the National Health Laboratory (NHL) in Yangon, Myanmar. These frozen samples were subsequently transported by an international courier from NHL to Niigata University, Japan, for further virological analyses to detect respiratory pathogens.

### 2.2. Detection of Respiratory Pathogens Using BioFire RP

To identify respiratory pathogens in RDT-negative specimens for influenza, SARS-CoV-2, or RSV, we tested these samples by using BioFire RP [32,33]. BioFire RP runs on BioFire^®^ FilmArray^®^ Systems (bioMérieux, Marcy-l’Étoile, France). We collected the results of pathogens detected by using BioFire RP. If multiple pathogens were detected in a single patient, each pathogen was counted separately. Therefore, the total number of pathogens detected exceeded the total number of patients. Patients in whom no pathogen was found were classified as “not detected”. The proportion of each pathogen was calculated and expressed as a percentage of the total number of pathogens detected. We extracted demographic data (sex and age) and clinical manifestations (fever, cough, runny nose, dyspnea, myalgia, arthralgia, nausea, and vomiting) from patients in whom only a single pathogen was detected by using the BioFire RP assay. Among these, only patients infected with RV/EV, RSV, or hMPV were included in the comparative analysis for the clinical symptoms, as these were the most commonly detected pathogens. Patients infected with other pathogens were excluded from this analysis due to the limited number of cases. Furthermore, cycle threshold (Ct) values could not be assessed because the BioFire^®^ RP panel is a qualitative assay that detects pathogen presence or absence without providing quantitative viral load information. We also focused on co-infections by comparing the clinical characteristics of patients simultaneously infected with both RV/EV and RSV to those with single infections of either virus. The clinical features were then compared across the three groups. Statistical analysis was performed using EZR version 1.54.23 [34]. Data were analyzed using χ^2^ and Kruskal–Wallis tests, and p values less than 0.05 were considered statistically significant. 

### 2.3. RV and EV Identification and Genotyping Using VP4/VP2 Genes

Since it is difficult to differentiate RVs and EVs with BioFire RP, RV/EV patient samples were separately analyzed by RT-PCR and genetic sequencing in this study. While the VP1 region is frequently utilized for EV/RV genotyping due to its high variability [30,35], its application is often limited by reduced sensitivity and PCR efficiency, particularly in samples with low viral load or degraded RNA [36]. To address these limitations, we employed a semi-nested RT-PCR protocol targeting the VP4/VP2 region, modified from the nested PCR method described by Wisdom et al. [31], which has demonstrated higher amplification efficacy and broader compatibility across diverse EV/RV types. All RV/EV-positive samples identified via the BioFire RP assay were subsequently subjected to RT-PCR and partial sequencing of the VP4/VP2 region to facilitate comprehensive phylogenetic analysis and accurate genotyping. The primer sequences employed are detailed in the Supplementary Materials (Table S1). To extract total viral RNA, 140 μL of each sample was processed using the QIAamp Viral RNA Mini Kit (Qiagen, Hilden, Germany), following the manufacturer’s guidelines. First-round PCR was performed using the PrimeScript High Fidelity One-Step RNA PCR Kit (TaKaRa Bio, Kusatsu, Shiga, Japan) with a forward primer of HEV/HRV OS and a reverse primer of HEV/HRV IAS. The initial PCR cycle must be warmed up for 30 min at 45 °C and activated at 95 °C for 2 min, followed by 40 cycles of 10 s at 98 °C for denaturation, 15 s at 50 °C for annealing, and 1 min at 68 °C for extension, concluding with an additional 5 min extension at 68 °C. The second round of PCR was performed using PrimeSTAR HS DNA Polymerase (TaKaRa Bio, Kusatsu, Shiga, Japan) with a forward primer of HEV/HRV IS and a reverse primer of HEV/HRV IAS. The PCR cycle entailed 2 min activation at 95 °C, followed by 30 cycles of 10 s at 98 °C for denaturation, 5 s at 50 °C for annealing, and 45 s at 72 °C for extension, concluding with an additional 7 min extension at 72 °C. The obtained amplified products were developed on 1.0% agarose gel. The PCR products of positive samples were subjected to Sanger sequencing, and the sequences were aligned using SeqMan Pro, Lasergene software (version 18.0.3) suite (DNASTAR, Inc., Madison, WI, USA) (Table S1). 

### 2.4. Phylogenetic Tree Analysis of RVs/EVs for Type Determination

To determine RV/EV types, phylogenetic analysis was performed using 137 reference sequences obtained from the GenBank database, based on representative strains listed in the 10th online report of the International Committee on Taxonomy of Viruses (ICTV) (https://ictv.global/report/chapter/picornaviridae/picornaviridae/enterovirus, accessed on 17 March 2025). A phylogenetic tree was constructed using MEGA 6 software with the neighbor-joining method [37]. Tree robustness was assessed by bootstrap analysis with 1,000 replicates. Genetic diversity, nucleotide sequence identity, and amino acid variations were further analyzed using BioEdit software 7.2.5 (http://www.mbio.ncsu.edu/BioEdit/, accessed on 16 March 2025). The nucleotide sequences obtained in this study have been deposited in the DNA Data Bank of Japan (DDBJ) under accession numbers [LC878070–LC878171].

## 3. Results

### 3.1. Detection of Respiratory Pathogens by BioFire RP

**Figure 1:**
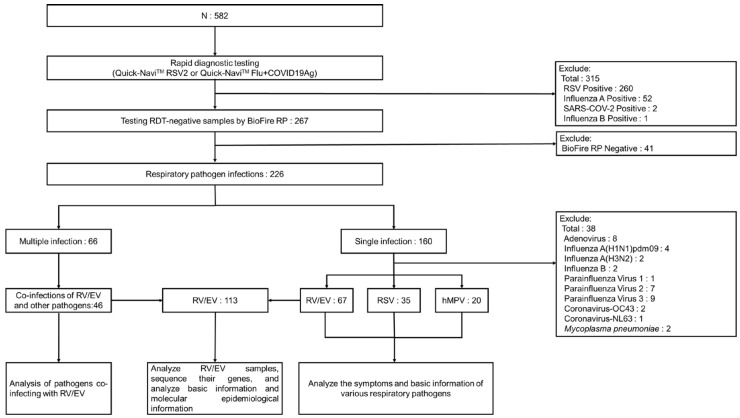
Data procedures. During the study period, we collected samples from 582 patients, and after RDT, 267 patients with negative RDT results participated in the study. We used multiplex PCR to examine the samples of these patients with negative RDT results, and a total of 299 pathogen infections were detected, of which 160 were single-pathogen infections and 66 were multiple-pathogen infections. We analyzed the symptoms and basic information of patients with single-pathogen infection. For patients with multiple infections, we analyzed the types of pathogens co-infected with RVs/EVs. In addition, we found that among all infected people, the number of patients infected with RVs/EVs was the largest, so we further analyzed the samples of patients infected with RVs/EVs.

During the study period of June–December 2023, a total of 582 patients with symptoms of acute respiratory infection were enrolled in the two medical institutions in Yangon, Myanmar. Among them, 260 patients who tested positive for RSV and 55 patients who tested positive for influenza or SARS-CoV-2 by rapid diagnostic tests (Quick-Navi™ RSV2 and Quick-Navi™ Flu+COVID19Ag, respectively) were excluded from this study. The remaining 267 patients who tested negative with rapid diagnostic tests were included in the final analysis (Figure 1).

All 267 patients were tested using the multiplex PCR assay, BioFire RP. Of these, 226 patients (84.6%) were positive for at least one respiratory pathogen, while 41 patients (15.4%) had no pathogens detected. Among the 226 positive cases, 160 (70.8%) had a single infection and 66 (29.2%) had multiple infections. In total, 299 pathogens were identified. The most frequently detected pathogen was RV/EV, accounting for 37.8% (113/299), followed by RSV at 22.4% (67), hMPV at 10.0% (30), and adenovirus at 7.7% (23) (Table 1).

**Figure 2:**
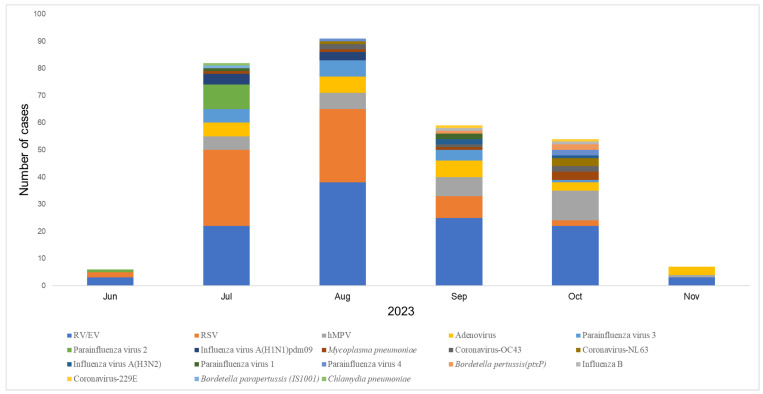
Epidemic curve of acute respiratory infections (ARIs) in Myanmar by month. We plotted the epidemic curve by month, which shows that the number of detected cases increased significantly from June, peaked in August, and then gradually decreased. RV/EV, RSV, and hMPV were most detected in August. Other pathogens, such as adenovirus, parainfluenza virus (types 1–4), Mycoplasma pneumoniae, and influenza A (H1N1) virus (pdm09), were also active from July to October.

The epidemic curve shows that the number of detected cases increased markedly from June, peaking in August, and gradually declined thereafter. RV/EV was consistently the most frequently detected pathogen across all months, with a peak in August (38 cases) (Figure 2). RSV showed a similar trend, with highest detection also in August (27 cases), followed by July and September. hMPV was detected sporadically, with the highest number in August (6 cases). Other pathogens such as adenovirus, parainfluenza viruses (types 1–4), *Mycoplasma pneumoniae*, and influenza A(H1N1)pdm09 also showed activity between July and October. Notably, influenza A(H1N1)pdm09 and *M. pneumoniae* were more commonly detected from July to September, while detections of Bordetella pertussis, coronaviruses (OC43, NL63, 229E), and influenza B remained low throughout the study period. These findings suggest that the co-circulation of multiple respiratory viruses was most intense during the rainy season months of July to September in Yangon.

### 3.2. Demographic and Clinical Characteristics of Patients Infected with RV/EV, RSV, and hMPV

Among the 160 patients with single-pathogen infections, 122 were infected with one of the three most common viruses: RV/EV in 67 patients (41.9%), RSV in 35 patients (21.9%), and hMPV in 20 patients (12.5%) (Table S2). We compared the demographic characteristics and clinical symptoms, including age, gender, fever, cough, rhinorrhea, dyspnea, myalgia, arthralgia, and nausea/vomiting, among patients infected with RV/EV, RSV, and hMPV (Table 2). Symptoms including headache (*n* = 2), sore throat (*n* = 2), diarrhea (*n* = 2), and sneezing (*n* = 1) were infrequently reported among patients with a single infection of RV/EV, RSV, or hMPV. Furthermore, no cases of fatigue, anosmia, and ageusia were documented within these groups. There were no significant differences in gender distribution (*p* = 0.18) or median age between the groups: 0.85 years in the RV/EV group, 1.0 year in the RSV group, and 2.0 years in the hMPV group (*p* = 0.11). Fever was significantly more frequent in the hMPV group (55.0%) compared to the RV/EV (22.4%) and RSV (22.9%) groups (*p* = 0.01). Cough and rhinorrhea were common in all groups, with no significant differences observed (*p* > 0.05). Dyspnea was more frequently observed in RV/EV-infected patients, but the difference was not statistically significant (*p* = 0.34). Other symptoms such as myalgia, nausea, and vomiting were uncommon across all groups and showed no significant differences (*p* > 0.05). 

### 3.3. Distribution of Single and Co-Infections Among RV/EV-Positive Patients

A total of 113 RV/EV cases were identified by using the BioFire RP assay (Figure 1). Among them, 67 patients (59.3%) had RV/EV as a single infection, while 46 patients (40.7%) were co-infected with one or more additional pathogens. Of the co-infected cases, 39 patients were co-infected with one other pathogen, and 7 patients had co-infections with two pathogens. The most common co-infecting pathogen was RSV, found in 22 cases (39.3%), followed by adenovirus in 9 cases (17.3%) and hMPV in 6 cases (11.5%). Other co-detected pathogens included *Mycoplasma pneumoniae*, *Bordetella pertussis* (*ptxP*), coronaviruses (NL63, 229E, OC43), parainfluenza viruses (1, 3, and 4), and *Chlamydia pneumoniae* (Table 3).

We compared the demographic and clinical characteristics of patients with RV/EV–RSV co-infections (*n* = 22) to those with single RV/EV or RSV infections (Table S3). Co-infections involving other viruses were excluded from analysis due to their limited occurrence (fewer than 10 cases). No statistically significant differences were identified among the three groups in terms of age, gender, or clinical symptoms, including fever, cough, rhinorrhea, dyspnea, myalgia, arthralgia, and nausea/vomiting. Although dyspnea was slightly more frequent in RV/EV single infections, but this difference was not statistically significant. 

### 3.4. Genotypes of RV/EV

**Figure 3:**
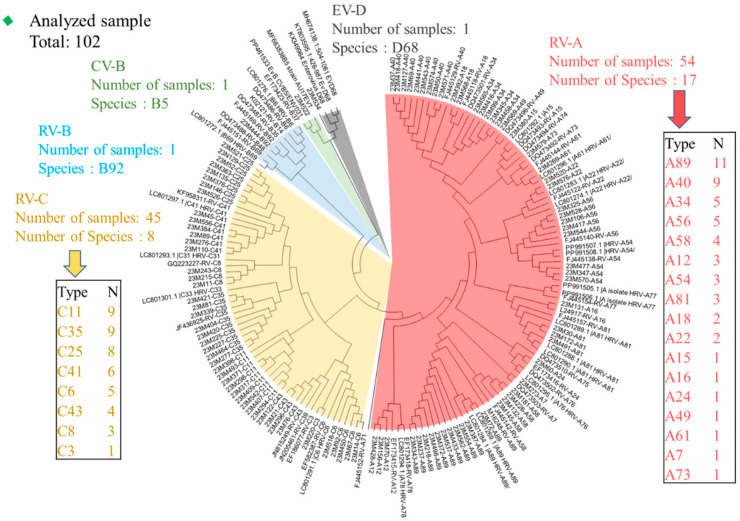
Phylogenetic analysis of the partial VP4/VP2 gene of rhinovirus. We marked RV-A in red, RV-B in blue, RV-C in yellow, CV-B in green, and EV-D in gray. A phylogenetic tree was constructed based on the 102 RV/EV sample sequences tested in this study and reference sequences containing genotype information from around the world. The tree was generated using the neighbor-joining method, and branch support was determined using 1000 bootstrap iterations using MEGA software. The generated tree nodes are annotated with bootstrap values.

Of the 113 RV/EV samples, 102 nucleotide sequences (90.26%) were successfully retrieved. Phylogenetic analysis of the VP4/VP2 region identified five species: RV-A, comprising 52.9% (54 cases); RV-B, representing 1.0% (1 case); RV-C, accounting for 44.1% (45 cases); Coxsackievirus (CV)-B, also at 1.0% (1 case); and EV-D, constituting 1.0% (1 case) of the positive samples (Figure 3).

In this study, RV-A was the most diverse species, encompassing 17 different types: RV-A7, A12, A15, A16, A18, A22, A24, A34, A40, A49, A54, A56, A58, A61, A73, A81, and A89 (Figure 3). Among these, the most prevalent genotypes were RV-A89 (11 cases), RV-A40 (9 cases), RV-A34 (5 cases), and RV-A20 (5 cases) (Table S4). Within the RV-B group, a single instance of RV-B5 was identified. Eight types of RV-C were observed for RV-C3, C6, C8, C11, C25, C35, C41, and C43. Among these, RV-C11 (nine cases), RV-C35 (nine cases), and RV-C25 (eight cases) were the most prevalent (Table S3). One CV-B (CV-B5) and one EV-D (EV-D68) were also detected within the EV species. A single case of EV-D68 infection was identified in a 9-month-old male outpatient who presented with fever, cough, and dyspnea. Another case involving CV-B5 infection was detected in a 4.6-year-old male outpatient whose symptoms included fever, cough, and rhinorrhea.

Next, we examined the seasonality of RVs/EVs based on the monthly distribution of each species during the study period. RVs were detected throughout the study period, with a peak in August, comprising 16 cases of RV-A and 22 cases of RV-C. RV-A was observed from June to October, peaking in July, while RV-C was detected from July to November, peaking in August. Additionally, CV-B was identified in September, and EV-D was detected in October (Figure S1).

## 4. Discussion

This study investigated the distribution and genetic characteristics of respiratory pathogens among outpatients with acute respiratory infections (ARIs) in Yangon, Myanmar, during 2023. By focusing on cases that were negative according to rapid diagnostic tests (RDTs) for influenza, RSV, and SARS-CoV-2, we identified a broader spectrum of circulating respiratory viruses using BioFire RP. The observation that patients initially tested negative for RSV and influenza via RDTs but were subsequently identified as positive using the BioFire RP assay underscores the limitation of RDTs in clinical practices. This discrepancy is likely attributable to the higher sensitivity, broader pathogen detection spectrum, and enhanced capability of BioFire RP to identify low viral loads [38,39]. In contrast, RDTs may fail to detect infections due to their lower sensitivity or if testing is performed outside the optimal antigen detection window [40,41]. These findings highlight the importance of utilizing multiplex PCR assays for more accurate diagnosis. RVs/EVs were the most frequently detected pathogens during the study period. While the demographic characteristics and most clinical symptoms were similar across RV/EV, RSV, and hMPV groups, fever was significantly more frequent in hMPV cases. Genotyping of RV/EV-positive samples revealed a total of 28 distinct genotypes, indicating a high level of genetic diversity. This study provides important insights into the epidemiology of respiratory infections in the outpatient setting in Myanmar during the post-COVID-19 era, addressing a gap in existing research in this region.

The results of this study show that RVs/EVs, RSV, and hMPV were the most frequently detected respiratory pathogens among children in Myanmar in 2023, particularly among those who tested negative with rapid diagnostic tests for influenza, SARS-CoV-2, and RSV. These findings align with global patterns observed in the post-COVID-19 period. A recent meta-analysis by Khales et al. examined the epidemiology of respiratory virus infections in children across studies conducted between 2020 and 2023, revealing that RVs/EVs and RSV were the most prevalent viruses, particularly among younger children and hospitalized patients [42]. The study emphasized the sustained circulation of RVs/EVs and the re-emergence of RSV as NPIs were lifted globally. Similarly, Zhao et al., using data from 92 sites worldwide, reported a consistent virus-specific time sequence in post-pandemic resurgences, with RVs resurging first, followed by seasonal coronavirus, parainfluenza virus, RSV, and hMPV, and influenza B resurging last [13]. Del Riccio et al. revealed that in tropical regions, the epidemic durations of certain viruses, such as hMPV and adenovirus, was reduced following the relaxation of non-pharmaceutical interventions, suggesting changes in transmission dynamics [11]. These findings indicate that the epidemiology of respiratory viruses has shifted in the post-pandemic era, and our findings from Myanmar add research context to our understanding of these changes in a tropical, outpatient population.

Indeed, limited reports on the epidemiology of respiratory pathogens are available so far from outpatient settings in Asia in 2023, particularly following the lifting of COVID-19-related restrictions. Comparisons with recent studies from Southern China and Taiwan provide a useful reference for interpreting our findings. In our outpatient cohort in Myanmar, the most frequently detected pathogens among RDT-negative patients were RVs/EVs (37.8%), RSV (22.4%), and hMPV (10.0%), followed by adenovirus and parainfluenza viruses. Similarly, studies in China during the same period reported RVs, RSV, and Mycoplasma pneumoniae (MP) as dominant pathogens. In a large inpatient cohort in Shenzhen, Wang et al. observed that HRV, MP, and RSV were the most prevalent pathogens in the post-pandemic period, although MP emerged more prominently than in our study, with unknown reason [43]. In Zhejiang, Li et al. also found that MP (28.6%), HRV (14.4%), and RSV (9.5%) were the leading causes of pediatric ARIs in 2023, with hMPV ranking lower than in our findings [44]. In contrast, a study from northern Taiwan conducted among hospitalized children using the same BioFire platform as in our study reported RSV (37.1%) as the most common pathogen, followed by RVs/EVs (21.5%) and parainfluenza virus 3 (17.1%), with multiple viral co-infections frequently observed [45]. These differences in pathogen detection rates may be influenced by several factors, including the timing and extent of NPI relaxation, population immunity, diagnostic practices, and geographic conditions. The relaxation of NPIs increases population exposure to circulating viruses, while reduced immune stimulation during prolonged NPI periods may contribute to an “immune debt,” making individuals more vulnerable to infection [46]. Viral factors (such as increased virulence, environmental stability, or changes in host susceptibility) may also play a role [47,48]. Climatic and regional factors may further shape the transmission dynamics of RSV and other respiratory pathogens [49]. Despite these variations, RVs/EVs and RSV consistently appeared as dominant pathogens across outpatient and inpatient settings in all three regions, underscoring their prominent role in post-pandemic respiratory infections.

Our long-term surveillance efforts in Myanmar have shown that the seasonality of respiratory viruses in this tropical setting is distinct from temperate climates. Influenza virus activity has consistently peaked during the rainy season, typically between June and August, as demonstrated by studies conducted between 2005 and 2015 [17,18]. More recent analyses during the COVID-19 pandemic indicated an atypical and delayed re-emergence of influenza A(H3N2) and B/Victoria between October and November 2021, following the decline in delta variant SARS-CoV-2 outbreaks [50]. Similarly, molecular epidemiological studies of RSV revealed a clear seasonal pattern, with annual peaks consistently observed from July to October [19,51]. Although less is known about the seasonality of other respiratory viruses in Myanmar, Kamata et al. analyzed hospitalized children with acute lower respiratory infections during 2017–2019 in Yangon and reported that RVs/EVs, adenovirus, and seasonal coronaviruses were detected year-round, with RVs/EVs especially prevalent during both the rainy and dry seasons [22]. Other studies in tropical countries have shown that the transmission of RVs/EVs has no obvious seasonality [52,53,54]. These findings suggest that while some viruses such as influenza and RSV follow clear monsoonal seasonality, others—particularly RVs/EVs—may circulate more continuously throughout the year, especially in pediatric populations. 

We compared the clinical symptoms of RV/EV, RSV, and hMPV infections among outpatients. Our findings showed that fever was significantly more common in patients with hMPV infection than in those infected with RVs/EVs or RSV, while other symptoms, such as cough and rhinorrhea, were similar across the three groups and showed no significant differences. This observation is consistent with previous studies reporting a higher frequency of fever in hMPV infections. For example, a 2022 study in Malaysia found that fever was more prevalent in children with hMPV compared to those with RSV [55]. Similarly, a multicenter study conducted in Japan from 2015 to 2017 among children under 3 years of age hospitalized for acute respiratory infections reported that hMPV-infected patients were more likely to present with high fever [56]. Another study involving high-risk children aged ≤24 months across 24 sites in the Northern Hemisphere between 2002 and 2006 also found that hMPV infections were associated with a higher frequency of fever upon admission [57]. Taken together, these findings—including our own—consistently demonstrate that hMPV is more frequently associated with febrile illness in children compared to other common respiratory viruses, such as EVs/RVs and RSV. In our study, we did not observe significant differences in symptom severity between patients with mono-infection and those with RV/EV–RSV co-infection. This may be attributed to the outpatient setting, where milder cases are typically encountered and hospitalization data were not available to assess clinical severity in detail. 

This study employed VP4/VP2 region sequencing to identify the circulating genotypes of RVs/EVs in Myanmar. Among the 102 RV/EV-positive samples successfully sequenced using the VP4/VP2 region, all three RV species were detected along with two enterovirus types—Coxsackievirus B and Enterovirus D68—representing a total of 28 distinct genotypes. Of these, RV-A was the most diverse, with 17 genotypes identified, followed by 8 genotypes of RV-C, and only a single genotype of RV-B. These findings are consistent with global reports from recent years showing that RV-A and RV-C are the predominant circulating species, while RV-B is infrequently detected [58,59,60,61]. In a surveillance study in Hainan Province, China, among 32 RV-positive samples from 2021 to 2023, RV-A accounted for 46.9%, RV-B accounted for 6.3%, and RV-C also accounted for 46.9% [58]. A study of respiratory samples in western Washington State, US, conducted between 2021 and 2022 showed that RV-A (59.0%) and RV-C (28.7%) predominated in 1,078 samples [59]. In addition, a study in Bulgaria from 2018 to 2023 showed that RV-A (15 cases) and RV-C (19 cases) were the main RV species in the region among 38 RV isolates, and RV-C became more prevalent after the COVID-19 epidemic [60]. Our study helps to fill a critical gap in the molecular epidemiology of RVs/EVs in Myanmar and contributes valuable data from a tropical, resource-limited setting. 

In this study, we identified one case of EV-D68 infection. EV-D68 is increasingly recognized as a re-emerging pathogen capable of causing severe respiratory illness, particularly in young children under five years of age. Clinical features often include hypoxia, wheezing, and, in severe cases, the need for intensive care or mechanical ventilation [62,63]. Beyond respiratory involvement, EV-D68 has also been associated with central nervous system complications, most notably acute flaccid myelitis (AFM), a rare but serious neurological condition [64,65]. Following its global resurgence in 2014, EV-D68 has been confirmed as a causative agent in both respiratory and neurologic disease clusters [66]. Although large-scale outbreaks of EV-D68 have been documented in the United States, Europe, and East Asia, data from Southeast Asia, including Myanmar, remain limited. Our pre-pandemic hospital-based study in Yangon detected EV-D68 in 42 of 570 children (7.4%) hospitalized with acute lower respiratory infections between 2017 and 2019, with most infections occurring in infants under 1 year of age and frequently presenting with cough, dyspnea, and wheezing [21]. Phylogenetic analysis from that study identified all circulating strains as belonging to clade B3, the globally dominant lineage since 2014. Although clade identification was not performed for our outpatient case, its detection underscores the continued presence of EV-D68 in Myanmar. Similarly, we identified one case of CV-B5 infection. CV-B5 has been associated with various clinical manifestations, such as aseptic meningitis [67], viral encephalitis, HFMD, and acute flaccid paralysis [29,68]. Although the CV-B5 case in our study presented with symptoms, including fever, cough, and rhinorrhea, the lack of hospitalization data limits our assessment to evaluate potential complications or the progression of illness.

This study has several limitations: First, the dataset was limited to patients from two outpatient clinics in Yangon and covered mainly the rainy season period from June to December 2023. This may affect the generalizability of our findings, as virus circulation patterns and healthcare-seeking behavior may vary across different regions, seasons, and populations. Additionally, factors such as access to healthcare services, illness severity, and willingness to undergo testing may have influenced the sample composition. To improve the robustness and representativeness of the findings, future studies should include data from multiple sites across Myanmar and extend surveillance over a full calendar year. Second, we did not include hospitalized patients, which may have led to underrepresentation of more severe respiratory infections. Third, the BioFire^®^ multiplex PCR assay used in this study does not detect all respiratory pathogens, including certain bacterial and fungal agents. Fourth, although we included influenza and RSV-negative cases according to rapid diagnostic tests (RDTs), they likely reflect low viral loads. While the clinical significance of low viral load remains uncertain, the symptoms of these patients may not fully represent typical RSV cases. Fifth, although this study detected EV-D68 and CV-B5 using VP4/VP2 region sequencing, it should be acknowledged that this genomic region is not regarded as the most definitive target for enterovirus typing. Therefore, the accuracy of genotype classification may be subject to certain limitations.

## 5. Conclusions

This study provides important insights into the post-COVID-19 epidemiology of respiratory pathogens in Myanmar, particularly during the 2023 rainy season. Our findings highlight the significant role of RVs/EVs, in addition to influenza and RSV, in contributing to the burden of acute respiratory infections among children in tropical, resource-limited settings. Despite historically receiving less attention, RVs/EVs are now recognized as major contributors to both upper and lower respiratory tract infections and, in some cases, systemic complications. These results underscore the urgent need to expand respiratory surveillance frameworks to include RVs/EVs and to strengthen laboratory capacity for their molecular detection and typing. Long-term, multicenter, and year-round surveillance—integrating both outpatient and inpatient data—is critical to monitoring viral trends, guide vaccine and diagnostic development, and inform timely public health responses. In the evolving landscape of respiratory virus circulation following the COVID-19 pandemic, comprehensive pathogen surveillance will be key to improving preparedness and response strategies across diverse global contexts.

## Figures and Tables

**Table 1:** Pathogen distribution (*n* = 299) detected by BioFire RP among RDT-negative 226 patients.

Pathogens	Number of Detections (%)
RV/EV	113 (37.8%)
RSV	67 (22.4%)
hMPV	30 (10.0%)
Adenovirus	23 (7.7%)
Parainfluenza virus 3	16 (5.4%)
Parainfluenza virus 2	10 (3.3%)
Influenza virus A(H1N1)pdm09	7 (2.3%)
*Mycoplasma pneumoniae*	6 (2.0%)
Coronavirus-OC43	5 (1.7%)
Coronavirus-NL63	4 (1.3%)
Influenza virus A(H3N2)	3 (1.0%)
Parainfluenza virus 1	3 (1.0%)
Parainfluenza virus 4	3 (1.0%)
*Bordetella pertussis* (*ptxP*)	3 (1.0%)
Influenza B	2 (0.7%)
Coronavirus-229E	2 (0.7%)
*Bordetella parapertussis* (*IS*1001)	1 (0.3%)
*Chlamydia pneumoniae*	1 (0.3%)

**Table 2:** The demographic and clinical characteristics of the three most prevalent pathogens (*n* = 122) among 226 patients infected with a single prevalent pathogen as determined by BioFire RP.

	RV/EV (*n* = 67)	RSV (*n* = 35) *	hMPV (*n* = 20)	*p*
Age (years) median (interquartile range)				
	0.85	1.0	2.0	0.42 ^b^
	(2.0, 5.0)	(0.7, 3.8)	(0.8, 3.5)	
Gender *n* (%)				0.18 ^c^
Male	37 (55.2) ^a^	22 (62.9)	8 (40.0%)	
Female	29 (43.3)	13 (37.1)	12 (60.0)	
Symptoms *n* (%)				
Fever (>37.5 °C)	15 (22.4)	8 (22.9)	11 (57.9)	0.01 ^c^
Cough	63 (94.0)	34 (97.1)	20 (100.0)	0.70 ^c^
Rhinorrhea	54 (80.6)	27 (77.1)	14 (73.7)	0.75 ^c^
Dyspnea	29 (43.3)	10 (28.6)	6 (31.6)	0.34 ^c^
Myalgia	2 (3.0)	4 (11.4)	0 (0.0)	0.15 ^c^
Arthralgia	2 (3.0)	4 (11.4)	0 (0.0)	0.15 ^c^
Nausea and Vomiting	2 (3.0)	2 (8.6)	2 (10.0)	0.48 ^c^

* These samples were negative for RSV-RDT. ^a^ One patient with RV/EV infection had unknown gender. ^b^ The median age of patients infected with the three pathogens was analyzed using the Kruskal–Wallis test. ^c^ The χ^2^ test was used to analyze the gender and symptoms of patients infected with the three pathogens.

**Table 3:** Proportions of pathogens in EV/RV co-infection detected by BioFire RP.

Pathogen	Cases (%)
RSV	22 (41.5%)
Adenovirus	9 (17.0%)
hMPV	6 (11.3%)
*Mycoplasma pneumoniae*	3 (5.7%)
Coronavirus NL63	3 (5.7%)
*Bordetella pertussis* (*ptxP*)	3 (5.7%)
Parainfluenza Virus 3	2 (3.8%)
Parainfluenza Virus 1	1 (1.9%)
Parainfluenza Virus 4	1 (1.9%)
Coronavirus 229E	1 (1.9%)
Coronavirus OC43	1 (1.9%)
*Chlamydia pneumoniae*	1 (1.9%)

Note: Among the 46 samples co-infected with RV/EV, 7 samples were co-infected with 2 pathogens, namely adenovirus and *Chlamydia pneumoniae*; hMPV and RSV; adenovirus and RSV; adenovirus and *Mycoplasma pneumoniae*; adenovirus and *Bordetella pertussis* (*ptxP*); coronavirus-OC43 and hMPV; and adenovirus and hMPV.

## Data Availability

The nucleotide sequences obtained in this study have been deposited in the DNA Data Bank of Japan (DDBJ) under accession numbers [LC878070–LC878171].

## Supplementary Materials

The following supporting information can be downloaded at https://www.mdpi.com/article/10.3390/v17060860/s1, Table S1. The primer used for the reverse transcriptase polymerase chain reaction (RT-PCR) of human enterovirus (HEV) and human rhinovirus (HRV); Table S2. Distribution of pathogens identified by BioFire^®^ FilmArray^®^ Respiratory Panel 2.1 among 160 patients with single-pathogen respiratory infections; Table S3. Distribution of rhinovirus and enterovirus species and types identified by VP4/VP2 sequencing among 102 successfully sequenced samples; Table S4. Demographic and clinical characteristics of patients infected with RSV and RV/EV alone compared with patients infected with RSV and RV/EV simultaneously; Figure S1. Monthly distribution of rhinovirus (RV) and enterovirus (EV) species identified by VP4/VP2 sequencing in Myanmar, 2023.

## References

[1] Cillóniz C., Pericàs J.M., Rojas J.R., Torres A. (2022). Severe Infections Due to Respiratory Viruses. Semin. Respir. Crit. Care Med..

[2] Moesker F.M., van Kampen J.J., van Rossum A.M., de Hoog M., Koopmans M.P., Osterhaus A.D., Fraaij P.L. (2016). Viruses as Sole Causative Agents of Severe Acute Respiratory Tract Infections in Children. PLoS ONE.

[3] Jones A.H., Ampofo W., Akuffo R., Doman B., Duplessis C., Amankwa J.A., Sarpong C., Sagoe K., Agbenohevi P., Puplampu N. (2016). Sentinel surveillance for influenza among severe acute respiratory infection and acute febrile illness inpatients at three hospitals in Ghana. Influenza Other Respir. Viruses.

[4] Lozano R., Naghavi M., Foreman K. (2012). Global and regional mortality from 235 causes of death for 20 age groups in 1990 and 2010: A systematic analysis for the Global Burden of Disease Study 2010. Lancet.

[5] Sharrow D., Hug L., You D., Alkema L., Black R., Cousens S., Croft T., Gaigbe-Togbe V., Gerland P., Guillot M. (2022). Global, regional, and national trends in under-5 mortality between 1990 and 2019 with scenario-based projections until 2030: A systematic analysis by the UN Inter-agency Group for Child Mortality Estimation. Lancet Glob. Health.

[6] Zhu H., Huang K., Han X., Pan Z., Cheng H., Wang Q., Wang Y., Sun W., Mi J., Yang T. (2025). The burden of acute respiratory infection in children under 5 attributable to economic inequality in low- and middle-income countries. BMJ Glob. Health.

[7] Tamerius J., Nelson M.I., Zhou S.Z., Viboud C., Miller M.A., Alonso W.J. (2011). Global influenza seasonality: Reconciling patterns across temperate and tropical regions. Env. Health Perspect..

[8] Bloom-Feshbach K., Alonso W.J., Charu V., Tamerius J., Simonsen L., Miller M.A., Viboud C. (2013). Latitudinal variations in seasonal activity of influenza and respiratory syncytial virus (RSV): A global comparative review. PLoS ONE.

[9] Lam T.T., Tang J.W., Lai F.Y., Zaraket H., Dbaibo G., Bialasiewicz S., Tozer S., Heraud J.M., Drews S.J., Hachette T. (2019). Comparative global epidemiology of influenza, respiratory syncytial and parainfluenza viruses, 2010–2015. J. Infect..

[10] Dallmeyer L.K., Schüz M.L., Fragkou P.C., Omony J., Krumbein H., Dimopoulou D., Dimopoulou K., Skevaki C. (2024). Epidemiology of respiratory viruses among children during the SARS-CoV-2 pandemic: A systematic review and meta-analysis. Int. J. Infect. Dis..

[11] Del Riccio M., Caini S., Bonaccorsi G., Lorini C., Paget J., van der Velden K., Meijer A., Haag M., McGovern I., Zanobini P. (2024). Global analysis of respiratory viral circulation and timing of epidemics in the pre-COVID-19 and COVID-19 pandemic eras, based on data from the Global Influenza Surveillance and Response System (GISRS). Int. J. Infect. Dis..

[12] Schüz M.L., Dallmeyer L., Fragkou P.C., Omony J., Krumbein H., Hünerbein B.L., Skevaki C. (2023). Global prevalence of respiratory virus infections in adults and adolescents during the COVID-19 pandemic: A systematic review and meta-analysis. Int. J. Infect. Dis..

[13] Zhao C., Zhang T., Guo L., Sun S., Miao Y., Yung C.F., Tomlinson J., Stolyarov K., Shchomak Z., Poovorawan Y. (2025). Characterising the asynchronous resurgence of common respiratory viruses following the COVID-19 pandemic. Nat. Commun..

[14] Savolainen-Kopra C., Korpela T., Simonen-Tikka M.-L., Amiryousefi A., Ziegler T., Roivainen M., Hovi T. (2012). Single treatment with ethanol hand rub is ineffective against human rhinovirus—Hand washing with soap and water removes the virus efficiently. J. Med. Virol..

[15] Takashita E., Shimizu K., Kawakami C., Momoki T., Saikusa M., Ozawa H., Kumazaki M., Usuku S., Tanaka N., Senda R. (2025). Impact of COVID-19 on Respiratory Virus Infections in Children, Japan, 2018–2023. Immun. Inflamm. Dis..

[16] Sudjaritruk T., Mueangmo O., Saheng J., Chaito T., Manowong S. (2025). P-1207. Resurgence of Respiratory Viral Infections Among Children Hospitalized in a Tertiary Care Hospital in Thailand After the COVID-19 Pandemic. Open Forum Infect. Dis..

[17] Dapat C., Saito R., Kyaw Y., Naito M., Hasegawa G., Suzuki Y., Dapat I.C., Zaraket H., Cho T.M., Li D. (2009). Epidemiology of human influenza A and B viruses in Myanmar from 2005 to 2007. Intervirology.

[18] Htwe K.T.Z., Dapat C., Shobugawa Y., Odagiri T., Hibino A., Kondo H., Yagami R., Saito T., Takemae N., Tamura T. (2019). Phylogeographic analysis of human influenza A and B viruses in Myanmar, 2010–2015. PLoS ONE.

[19] Phyu W.W., Htwe K.T.Z., Saito R., Kyaw Y., Lin N., Dapat C., Osada H., Chon I., Win S.M.K., Hibino A. (2021). Evolutionary analysis of human respiratory syncytial virus collected in Myanmar between 2015 and 2018. Infect. Genet. Evol..

[20] Tachikawa J., Aizawa Y., Kobayashi T., Ikuse T., Kamata K., Win S.M.K., Di Ja L., Thein K.N., Win N.C., Thida A. (2023). Detection of parechovirus-A in hospitalized children with acute lower respiratory infection in Myanmar, 2017–2018. J. Med. Virol..

[21] Ikuse T., Aizawa Y., Kachikawa R., Kamata K., Osada H., Win S.M.K., Di Ja L., Win N.C., Thein K.N., Thida A. (2024). Detection of enterovirus D68 among children with severe acute respiratory infection in Myanmar. J. Microbiol. Immunol. Infect..

[22] Kamata K., Thein K.N., Di Ja L., Win N.C., Win S.M.K., Suzuki Y., Ito A., Osada H., Chon I., Phyu W.W. (2022). Clinical manifestations and outcome of viral acute lower respiratory infection in hospitalised children in Myanmar. BMC Infect. Dis..

[23] Yamashita S., Ikegame S., Nakatomi K., Sakurai Y., Shuto H., Sato N., Mizoguchi Y., Uehara M., Nakashima N., Okamoto I. (2023). Respiratory Virus Infections during the COVID-19 Pandemic Revealed by Multiplex PCR Testing in Japan. Microbiol. Spectr..

[24] Popowitch E.B., Kaplan S., Wu Z., Tang Y.W., Miller M.B. (2022). Comparative Performance of the Luminex NxTAG Respiratory Pathogen Panel, GenMark eSensor Respiratory Viral Panel, and BioFire FilmArray Respiratory Panel. Microbiol. Spectr..

[25] Tapparel C., Siegrist F., Petty T.J., Kaiser L. (2013). Picornavirus and enterovirus diversity with associated human diseases. Infect. Genet. Evol..

[26] Jacobs Samantha E., Lamson Daryl M., St. George K., Walsh Thomas J. (2013). Human Rhinoviruses. Clin. Microbiol. Rev..

[27] Savolainen C., Blomqvist S., Hovi T. (2003). Human rhinoviruses. Paediatr. Respir. Rev..

[28] Royston L., Tapparel C. (2016). Rhinoviruses and Respiratory Enteroviruses: Not as Simple as ABC. Viruses.

[29] Machado R.S., Tavares F.N., Sousa I.P. (2024). Global landscape of coxsackieviruses in human health. Virus Res..

[30] McIntyre C.L., Knowles N.J., Simmonds P. (2013). Proposals for the classification of human rhinovirus species A, B and C into genotypically assigned types. J. Gen. Virol..

[31] Wisdom A., Leitch E.C.M., Gaunt E., Harvala H., Simmonds P. (2009). Screening Respiratory Samples for Detection of Human Rhinoviruses (HRVs) and Enteroviruses: Comprehensive VP4-VP2 Typing Reveals High Incidence and Genetic Diversity of HRV Species C. J. Clin. Microbiol..

[32] Leber A.L., Everhart K., Daly J.A., Hopper A., Harrington A., Schreckenberger P., McKinley K., Jones M., Holmberg K., Kensinger B. (2018). Multicenter Evaluation of BioFire FilmArray Respiratory Panel 2 for Detection of Viruses and Bacteria in Nasopharyngeal Swab Samples. J. Clin. Microbiol..

[33] Eckbo E.J., Locher K., Caza M., Li L., Lavergne V., Charles M. (2021). Evaluation of the BioFire(R) COVID-19 test and Respiratory Panel 2.1 for rapid identification of SARS-CoV-2 in nasopharyngeal swab samples. Diagn. Microbiol. Infect. Dis..

[34] Kanda Y. (2013). Investigation of the freely available easy-to-use software ‘EZR’ for medical statistics. Bone Marrow Transpl..

[35] Harvala H., Broberg E., Benschop K., Berginc N., Ladhani S., Susi P., Christiansen C., McKenna J., Allen D., Makiello P. (2018). Recommendations for enterovirus diagnostics and characterisation within and beyond Europe. J. Clin. Virol..

[36] Kitamura K., Arita M. (2024). Evaluation of VP4-VP2 sequencing for molecular typing of human enteroviruses. PLoS ONE.

[37] Tamura K., Stecher G., Peterson D., Filipski A., Kumar S. (2013). MEGA6: Molecular Evolutionary Genetics Analysis Version 6.0. Mol. Biol. Evol..

[38] Gosert R., Koller R., Meyer J., Dräger S., Ramette A., Bingisser R., Nickel C.H., Bassetti S., Sutter S.T., Keller P.M. (2024). Multicenter Evaluation of the QIAstat-Dx and the BioFire Multiplex Panel Tests for the Detection of Respiratory Pathogens. J. Med. Virol..

[39] Creager H.M., Cabrera B., Schnaubelt A., Cox J.L., Cushman-Vokoun A.M., Shakir S.M., Tardif K.D., Huang M.-L., Jerome K.R., Greninger A.L. (2020). Clinical evaluation of the BioFire® Respiratory Panel 2.1 and detection of SARS-CoV-2. J. Clin. Virol..

[40] Tamura D., Morisawa Y., Mato T., Nunomiya S., Yoshihiro M., Maehara Y., Ito S., Ochiai Y., Yamagishi H., Tajima T. (2024). Temporal Trend of the SARS-CoV-2 Omicron Variant and RSV in the Nasal Cavity and Accuracy of the Newly Developed Antigen-Detecting Rapid Diagnostic Test. Diagnostics.

[41] Zou X., Chang K., Wang Y., Li M., Zhang W., Wang C., Lu B., Xiong Z., Han J., Zhang Y. (2019). Comparison of the Cepheid Xpert Xpress Flu/RSV assay and commercial real-time PCR for the detection of influenza A and influenza B in a prospective cohort from China. Int. J. Infect. Dis..

[42] Khales P., Razizadeh M.H., Ghorbani S., Moattari A., Saadati H., Tavakoli A. (2025). Prevalence of respiratory viruses in children with respiratory tract infections during the COVID-19 pandemic era: A systematic review and meta-analysis. BMC Pulm. Med..

[43] Wang H., Guo Y., Wang R., Liu Z., Li L., Li Y., Bao Y., Wang W. (2025). Epidemiological Shifts in Children Respiratory Pathogens in Shenzhen, China: A Comparative Analysis Before and After the Relaxation of COVID-19 Non-Pharmaceutical Interventions. Influenza Other Respir. Viruses.

[44] Li H., Yang Y., Tao R., Shang S. (2024). Analyzing infections caused by 11 respiratory pathogens in children: Pre- and post-COVID-19 pandemic trends in China. J. Med. Virol..

[45] Chen C.-L., Chen Y.-C., Hsiao H.-L., Chang Y.-J., Li H.-C., Aydin M.A., Chiu C.-H. (2025). Multiple viral infections and antimicrobial use in hospitalized children with respiratory illness during pandemic and early post-pandemic era, Taiwan. Heliyon.

[46] Hatter L., Eathorne A., Hills T., Bruce P., Beasley R. (2021). Respiratory syncytial virus: Paying the immunity debt with interest. Lancet Child. Adolesc. Health.

[47] Nenna R., Pierangeli A., Matera L., Petrarca L., Conti M.G., Mancino E., di Mattia G., La Regina D.P., Virgili F., Papoff P. (2024). Respiratory Syncytial Virus Bronchiolitis Before and After COVID-19 Pandemic: Has the Immunity Debt Been Paid Off?. Pediatr. Infect. Dis. J..

[48] Pierangeli A., Nenna R., Fracella M., Scagnolari C., Oliveto G., Sorrentino L., Frasca F., Conti M.G., Petrarca L., Papoff P. (2023). Genetic diversity and its impact on disease severity in respiratory syncytial virus subtype-A and -B bronchiolitis before and after pandemic restrictions in Rome. J. Infect..

[49] Baker R.E., Mahmud A.S., Wagner C.E., Yang W., Pitzer V.E., Viboud C., Vecchi G.A., Metcalf C.J.E., Grenfell B.T. (2019). Epidemic dynamics of respiratory syncytial virus in current and future climates. Nat. Commun..

[50] Chon I., Saito R., Kyaw Y., Aye M.M., Setk S., Phyu W.W., Wagatsuma K., Li J., Sun Y., Otoguro T. (2023). Whole-Genome Analysis of Influenza A(H3N2) and B/Victoria Viruses Detected in Myanmar during the COVID-19 Pandemic in 2021. Viruses.

[51] Li J., Chon I., Phyu W.W., Kyaw Y., Aye M.M., Setk S., Win S.M.K., Yoshioka S., Wagatsuma K., Sun Y. (2025). Molecular epidemiological surveillance of respiratory syncytial virus infection in Myanmar from 2019 to 2023. Sci. Rep..

[52] Snoeck C.J., Evdokimov K., Xaydalasouk K., Mongkhoune S., Sausy A., Vilivong K., Pauly M., Hübschen J.M., Billamay S., Muller C.P. (2021). Epidemiology of acute respiratory viral infections in children in Vientiane, Lao People’s Democratic Republic. J. Med. Virol..

[53] Etemadi M.R., jalilian F.A., Othman N., Lye M.-S., Ansari S., Yubbu P., Sekawi Z. (2019). Diversity of respiratory viruses detected among hospitalized children with acute lower respiratory tract infections at Hospital Serdang, Malaysia. J. Virol. Methods.

[54] Chong Y.M., Chan Y.F., Jamaluddin M.F.H., Hasan M.S., Pang Y.K., Ponnampalavanar S., Syed Omar S.F., Sam I.C. (2022). Rhinovirus/enterovirus was the most common respiratory virus detected in adults with severe acute respiratory infections pre-COVID-19 in Kuala Lumpur, Malaysia. PLoS ONE.

[55] Ng D.C.-E., Liew C.-H., Tan K.K., Awang E.H.b., Nazri F.N.b.A., Maran A.K.T., Mohan V.A.a.l.C., Ramachandran D., Chok M., Teh C.H. (2024). Clinical comparison of HMPV and RSV infections in hospitalised Malaysian children: A propensity score matched study. Clin. Respir. J..

[56] Taniguchi A., Kawada J.-i., Go K., Fujishiro N., Hosokawa Y., Maki Y., Sugiyama Y., Suzuki M., Tsuji T., Hoshino S. (2019). Comparison of Clinical Characteristics of Human Metapneumovirus and Respiratory Syncytial Virus Infections in Hospitalized Young Children. Jpn. J. Infect. Dis..

[57] Anderson E.J., Simões E.A.F., Buttery J.P., Dennehy P.H., Domachowske J.B., Jensen K., Lieberman J.M., Losonsky G.A., Yogev R. (2012). Prevalence and Characteristics of Human Metapneumovirus Infection Among Hospitalized Children at High Risk for Severe Lower Respiratory Tract Infection. J. Pediatr. Infect. Dis. Soc..

[58] Xiao M., Banu A., Jia Y., Chang M., Wang G., An J., Huang Y., Hu X., Tang C., Li Z. (2024). Circulation pattern and genetic variation of rhinovirus infection among hospitalized children on Hainan Island, before and after the dynamic zero-COVID policy, from 2021 to 2023. J. Med. Virol..

[59] Goya S., Wendm S.T., Xie H., Nguyen T.V., Barnes S., Shankar R.R., Sereewit J., Cruz K., Pérez-Osorio A.C., Mills M.G. (2024). Genomic Epidemiology and Evolution of Rhinovirus in Western Washington State, 2021–2022. J. Infect. Dis..

[60] Georgieva I., Stoyanova A., Angelova S., Korsun N., Stoitsova S., Nikolaeva-Glomb L. (2023). Rhinovirus Genotypes Circulating in Bulgaria, 2018–2021. Viruses.

[61] Baillie V.L., Moore D.P., Mathunjwa A., Morailane P., Simões E.A.F., Madhi S.A. (2019). Molecular Subtyping of Human Rhinovirus in Children from Three Sub-Saharan African Countries. J. Clin. Microbiol..

[62] Grizer C.S., Messacar K., Mattapallil J.J. (2024). Enterovirus-D68—A Reemerging Non-Polio Enterovirus that Causes Severe Respiratory and Neurological Disease in Children. Front. Virol..

[63] Sooksawasdi Na Ayudhya S., Laksono B.M., van Riel D. (2021). The pathogenesis and virulence of enterovirus-D68 infection. Virulence.

[64] Esposito S., Chidini G., Cinnante C., Napolitano L., Giannini A., Terranova L., Niesters H., Principi N., Calderini E. (2017). Acute flaccid myelitis associated with enterovirus-D68 infection in an otherwise healthy child. Virol. J..

[65] Messacar K., Abzug M.J., Dominguez S.R. (2016). The Emergence of Enterovirus-D68. Microbiol. Spectr..

[66] Imamura T., Oshitani H. (2015). Global reemergence of enterovirus D68 as an important pathogen for acute respiratory infections. Rev. Med. Virol..

[67] Shiohama T., Omata T., Muta K., Kodama K., Fujii K., Shimojo N. (2016). Focal Coxsackie virus B5 encephalitis with synchronous seizure cluster and eruption: Infantile case. Pediatr. Int..

[68] Tan M., Suo J., Zhang Z., He W., Tan L., Jiang H., Li M., He J., Pan Y., Xu B. (2023). Molecular characterization of coxsackievirus B5 from the sputum of pneumonia children patients of Kunming, Southwest China. Virol. J..

