# Supplementary material for: Surveillance of Respiratory Pathogens Among Rapid Diagnostic Test-Negative Acute Respiratory Infection Patients in Myanmar in 2023, with a Focus on Rhinovirus and Enterovirus Genotyping
Source: Viruses. 2025 Jun 17;17(6):860. doi: 10.3390/v17060860 (PMC12197359; doi:10.3390/v17060860)

Table S1. The primer used for reverse transcriptase polymerase chain reaction (RT-PCR) of human enterovirus (HEV) and human rhinovirus (HRV).

Primer	Application	Sequence (5’ to 3’)
HEV/HRV OS	one-step RT-PCR	CCGGCCCCTGAATGYGGCTAA
HEV/HRV IAS	one-step RT-PCR<br>second PCR<br>sequencing	TCWGGHARYTTCCAMCACCANCC
HEV/HRV IS	second PCR<br>sequencing	ACCRACTACTTTGGGTGTCCGTG

Abbreviations: OS, outer sense; IAS, inner antisense; IS, inner sense.

Table S2. Distribution of pathogens identified by BioFire® FilmArray® Respiratory Panel 2.1 among 160 patients with single-pathogen respiratory infections.

**Pathogens**	**Number of detections (%)**
Rhinovirus and Enterovirus	67 (41.9%)
Respiratory Syncytial Virus	35 (21.9%)
Human Metapneumovirus	20 (12.5%)
Parainfluenza Virus 3	9 (5.6%)
Adenovirus	8 (5.0%)
Parainfluenza Virus 2	7 (4.4%)
Influenza A H1-2009	4 (2.5%)
Mycoplasma pneumoniae	2 (1.3%)
Influenza A H3	2 (1.3%)
Influenza B	2 (1.3%)
Coronavirus OC43	2 (1.3%)
Coronavirus NL63	1 (0.6%)
Parainfluenza Virus 1	1 (0.6%)

Table S3. Demographic and clinical characteristics of patients with singular RV/EV and RSV infections compared to those with concurrent RV/EV and RSV co-infections.

	**RV/EV (n=67)**	**RSV (n=35)***	**RV/EV & RSV (n=22)**	**p-value**
Age (years)				
Median (interquartile range)	0.9 (2.0, 5.0)	1.0 (0.7, 3.8)	1.0 (0.7, 3.0)	0.17 [a]
Gender, n (%)				
Male	37	22	13	0.68 [b]
Female	29	13	9	
Symptoms, n (%)				
Fever (≥37.5°C)	15 (22.4)	8 (22.9)	4(18.2)	0.92 [b]
Cough	63 (94.0)	34 (97.1)	21(95.5)	0.86 [b]
Rhinorrhea	54 (80.6)	27 (77.1)	18(81.8)	0.91 [b]
Dyspnea	29 (43.3)	10 (28.6)	4(18.2)	0.23 [b]
Myalgia	2 (3.0)	4 (11.4)	1(4.5)	0.24 [b]
Arthralgia	2 (3.0)	4 (11.4)	1(4.5)	0.24 [b]
Nausea and Vomiting	2 (3.0)	2 (8.6)	2(9.1)	0.15 [b]

*These samples were negative for RSV-RDT.

[a]The Kruskal−Wallis test was employed to compare the median age across three patient groups.

[b]The $\chi 2$ test was used to analyze the gender and symptoms of patients in three groups.

Table S4. Distribution of rhinovirus and enterovirus species and types identified by VP4/VP2 sequencing among 102 successfully sequenced samples.

Species	Type	Number of samples
**Rhinovirus (RV)**		
Species A (54)*	A89	11
	A40	9
	A34	5
	A56	5
	A58	4
	A12	3
	A54	3
	A81	3
	A18	2
	A22	2
	A15	1
	A16	1
	A24	1
	A49	1
	A61	1
	A7	1
	A73	1
Species B (1)*	B92	1
Species C (8)*	C11	9
	C35	9
	C25	8
	C41	6
	C6	5
	C43	4
	C8	3
	C3	1
**Coxsackievirus (CV)**		

Species B (1)*	B5	1
**Enterovirus (EV)**		
Species D (1)*	D68	1

*The number in parentheses indicates the total number of sequences classified under each species.

Figure S1. Monthly distribution of rhinovirus (RV) and enterovirus (EV) species identified by VP4/VP2 sequencing in Myanmar, 2023. This figure illustrates the monthly detection of RV-A, RV-B, RV-C, CV-B, and EV-D among outpatient respiratory samples collected between June and November 2023. RV-A and RV-C were the most frequently detected species throughout the study period, with a peak in August. CV-B and EV-D were each detected in a single case during September and October, respectively.

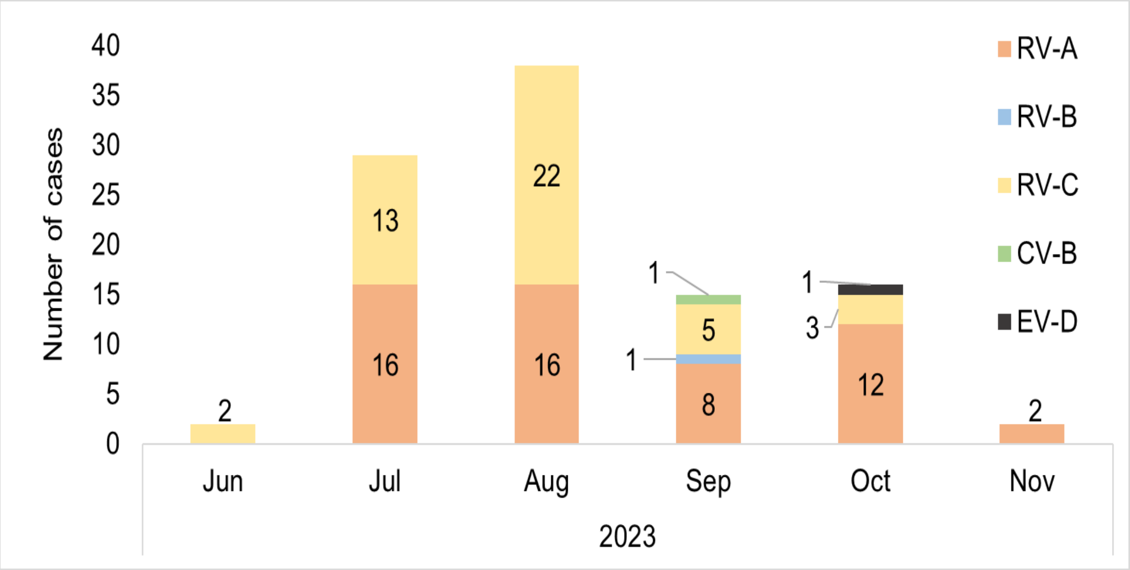

Supplement: Supplementary file 1 [file viruses-17-00860-s001.zip › viruses-3662092-supplementary.pdf]
